# Effect of environmental conditions on two vibriophages and their host

**DOI:** 10.3389/fmicb.2026.1914226

**Published:** 2026-10-01

**Authors:** Marcela León, Neri Vargas, Kívely Lozano, Santiago Gacitúa, Carlos Omar Lomelí-Ortega, Irais Ramírez-Sánchez, Eduardo Quiroz-Guzmán, Roberto Bastías

**Affiliations:** 1 Instituto de Biología, Pontificia Universidad Católica de Valparaíso, Valparaíso, Chile; 2 Centro de Investigaciones Biológicas del Noroeste S.C, La Paz, Mexico

**Keywords:** bacteriophage, environmental performance, *Vibrio*, *Vibrio anguillarum*, *Vibrio parahaemolyticus*, vibriophages

## Abstract

Bacteriophages play a key role in regulating *Vibrio* populations in marine environments, and environmental conditions may influence their replication and interactions with bacterial hosts. This study aimed to isolate and characterize the bacteriophage PvVp2, which infects the pandemic *Vibrio parahaemolyticus* strain KX, and to compare its environmental performance with that of phage CHOED, a lytic phage infecting *Vibrio anguillarum*. Phage PvVp2 possesses a 43,158 bp double-stranded DNA genome encoding a MazG-like pyrophosphatase, a putative auxiliary metabolic gene. Phylogenetic analysis clustered PvVp2 with vibriophages vB_Vc_SrVc2 and vB_Vc_SrVc9, sharing nearly 100% nucleotide identity. To evaluate the effect of environmental conditions on phage–host interactions, the replication of both phages was assessed under different temperatures, pH values and salinities. Performance curves showed that *V. parahaemolyticus* and PvVp2 were adapted to warmer conditions than *V. anguillarum* and CHOED, respectively. PvVp2 replicated efficiently between 22 °C and 40 °C, whereas CHOED replication was restricted to 14 °C–30 °C. Both phages replicated over narrower ranges of environmental conditions than their bacterial hosts, and deviations from optimal conditions increased latent periods and reduced burst sizes. Infection assays further showed that CHOED displayed greater lytic activity at 14 and 25 °C, whereas PvVp2 exhibited a lower but relatively stable lytic activity across the temperatures evaluated. These findings demonstrate that temperature, pH and salinity strongly influence phage replication and phage–host interactions, highlighting that the environmental range supporting phage replication is generally narrower than that supporting bacterial growth.

## Introduction

Vibrios are among the most abundant bacterial groups in marine environments. Their populations can be influenced by changes in environmental conditions such as temperature, salinity, and pH (Nishiguchi, 2000; Eiler et al., 2006; Vezzulli et al., 2015). In fact, some researchers have proposed vibrios as true barometers of climate change occurring in the oceans (Baker-Austin et al., 2017).

In this context, it is essential to understand how changes in environmental conditions can impact the populations of these bacteria. This is especially relevant considering that several *Vibrio* species can be pathogenic to humans (Baker-Austin et al., 2018) or marine animals (Mohamad et al., 2019; Sanches-Fernandes et al., 2022). *Vibrio parahaemolyticus* is a Gram-negative bacterium, abundant in estuaries and a leading cause of gastroenteritis associated with the consumption of raw or undercooked shellfish. Its main virulence factors include two type III secretion systems (T3SS-1 and T3SS-2), a thermostable direct hemolysin (TDH), and a TDH-related hemolysin (TRH; Wang et al., 2015). In 1995, a clone of this species caused the first pandemic attributed to this strain, reaching areas as remote as southern Chile (García et al., 2013). In recent years, some studies in Europe have reported outbreaks of *V. parahaemolyticus* in areas where it had not been previously detected, possibly associated with extreme weather events and elevated sea temperatures (Baker-Austin et al., 2013, 2016). A similar explanation was proposed for the spread of the pandemic clone of *V. parahaemolyticus* along the Pacific coast of South America, which may have been related to the El Niño phenomenon (Martinez-Urtaza et al., 2016).

These findings suggest that changes in environmental conditions could alter *Vibrio* populations in the environment. However, these bacterial communities are also affected by the presence of their natural predators, bacteriophages (Chevallereau et al., 2022). In marine environments, bacteriophages can directly influence the structure of bacterial communities and their evolution (Breitbart et al., 2018). Furthermore, phage infectivity can also be altered by changes in environmental conditions (Tokman et al., 2016; Greenrod et al., 2025).

Numerous bacteriophages capable of infecting different *Vibrio* species have been isolated (Bastías et al., 2010; Higuera et al., 2013; Kim et al., 2020; Lomelí-Ortega et al., 2023; Arce et al., 2024). In most cases, these bacteriophages are isolated and characterized for use as potential biological control agents (Higuera et al., 2013; Quiroz-Guzmán et al., 2018; González-Gómez et al., 2023). In this regard, one of the species of greatest interest, against which several bacteriophages have been isolated, is *Vibrio anguillarum*. This bacterium is capable of infecting more than 50 marine species, including some of economic importance to aquaculture (Frans et al., 2011). Despite numerous studies, very few have examined how environmental conditions affect interactions between vibrios and their bacteriophages, and often they focus on the impact of these variables on the efficacy of phage therapy (Silva et al., 2014). To fully understand this phenomenon, it is necessary to begin with basic aspects, such as determining the environmental conditions under which vibriophages can replicate in their host bacteria.

In this study, we report the isolation of a new phage capable of infecting the pandemic strain of *V. parahaemolyticus*. To understand how environmental conditions can affect the interaction between this phage and its bacterial host, we evaluated the performance curves of both the bacteria and the phage under different temperatures, salinities, and pH levels. We compared these interactions with those involving phage CHOED (León et al., 2019), which is specific to *V. anguillarum* and adapted to slightly colder environments than *V. parahaemolyticus* (Lovell, 2017; Hickey and Lee, 2018; Brumfield et al., 2023). Finally, we assessed how these environmental factors also impact the replication parameters of these phages, providing insights into how environmental conditions influence their replication cycles. This work serves as a starting point for understanding how changing environmental conditions could affect the interactions between vibrios and their phages, with potential implications for *Vibrio* population dynamics in marine ecosystems.

## Materials and methods

### Bacterial strain, bacteriophages and culture media

The *V. anguillarum* PF4 strain (serotype O3) used in this study was initially isolated from an aquaculture center in southern Chile (Silva-Rubio et al., 2008). The CHOED bacteriophage that naturally infects the *V. anguillarum* PF4 strain is a lytic phage isolated from mollusks in southern Chile (Higuera et al., 2013). The *Vibrio parahaemolyticus* KX strain corresponds to the pandemic clone O3:K6 that caused a pandemic between 1996 and 2010 (García et al., 2013). The phage PvVp2 that infects the *V. parahaemolyticus* KX is a lytic phage isolated in this study from Baja California Sur, Mexico.

The bacteria were grown in synthetic seawater (23.4 g/L NaCl, 24.7 g/L MgSO_4_ × 7H_2_O, 1.5 g/L KCl, and 1.43 g/L CaCl_2_ × 2H_2_O) supplemented with 1% Bactotryptone (Gifco) and 0.5% yeast extract, unless otherwise indicated. This medium was supplemented with 1.5% agar for solid culture.

### Phage isolation and genome analysis

Phage DNA was extracted using the phenol-chloroform protocol (Sambrook and Russell, 2006). Briefly, 1 mL of phage stock solution was transferred to a microcentrifuge tube and treated with 5 μL of DNase I and 5 μL of RNase A (10 mg/mL) at 37 °C for 60 min. Subsequently, 50 μL of 10% SDS, 100 μL of 0.2 M EDTA, and 2.5 μL of proteinase K (20 mg/mL) were added, followed by incubation at 56 °C for 90 min. After incubation, 300 μL of phenol was added, the sample was mixed thoroughly and centrifuged at 3,500 × g for 10 min. The aqueous phase was carefully collected without disturbing the interphase, transferred to a new microtube, and extracted again with an equal volume of phenol-chloroform (1:1, v/v). A final chloroform extraction was performed to remove any residual phenol. The resulting aqueous phase was mixed with 200 μL of 3 M sodium acetate and 200 μL of cold absolute ethanol, then incubated on ice for 30 min. Following incubation, the mixture was centrifuged at 15,000 × *g* for 30 min. The supernatant was discarded, and the pellet was washed three times with 70% cold ethanol, air-dried at room temperature, and resuspended in 25 μL of nuclease-free water.

The quality of the extracted DNA was assessed by electrophoresis on a 1% agarose gel at 90 V for 40 min. DNA bands were visualized under ultraviolet light using a transilluminator. The migration pattern of the bands was used to evaluate DNA integrity and purity. DNA concentration and purity were further determined using a NanoDrop spectrophotometer.

Phage genomes were sequenced using the Illumina platform. The FASTQ files were uploaded to the Galaxy CPT Public platform^1^ (Ramsey et al., 2020), where a standard bacteriophage assembly workflow, “Phage Genome Assembler v2021.01,” was used. Contigs were generated using different k-mer sizes, and their lengths were compared among the three k-mers employed in the assembly. Since all k-mer values produced a single contig of very similar length, the contig with an average coverage representative of the three results was selected for subsequent analyses.

The genome annotation was initially conducted with Prokka (Seemann, 2014) and further refined through manual curation based on BLASTp searches against closely related reference phages; specifically, Vibrio phage F23s2 (ON080941.1) and Vibrio phage vB_Vc_SrVc9 (LR794124.1) were used for this purpose. The annotated genome was examined using Proksee (Grant et al., 2023) to assess gene content and G+C composition. The presence of virulence or antimicrobial resistance determinants was evaluated using the CARD Resistance Gene Identifier tool (Alcock et al., 2023). Transfer RNA genes were predicted with tRNAscan-SE v2.0 (Chan et al., 2021). Taxonomic classification was inferred through the construction of viral proteomic trees using the ViPTree web server hosted by GenomeNet (Nishimura et al., 2017) and confirmed with taxMyPhage (Millard et al., 2025), whereas whole-genome alignments were visualized with the DiGAlign viewer (Nishimura et al., 2024). In both analyses, complete phage genome sequences were employed under default parameters. Phage termini was evaluated with the tool PhageTerm (Garneau et al., 2017).

### Growth curve

To understand how environmental conditions affect the growth of the two *Vibrio*, we evaluated the effects of temperature, pH, and salinity, three key physicochemical factors that vary in marine environments and influence bacterial physiology. The Temperatures used in this assay were 4 °C, 10 °C, 14 °C, 22 °C, 25 °C, 30 °C, 37 °C, and 40 °C. For *Vibrio parahaemolyticus*, a temperature of 45°C was also included. Salinities used were 0%, 0.85%, 2%, 3.5%, 5%, 7%, and 10% p/v, and pH values were 3, 4, 5, 6, 7, 7.7, 8, and 9. To assess the independent effect of each variable, only one parameter was varied in each experiment, with all other culture conditions held constant. Temperature assays were conducted at pH 7 and 3.5% (w/v) salinity; pH assays at 25 °C and 3.5% (w/v) salinity; and salinity assays at 25 °C and pH 7.

In brief, the strains were cultured in parallel using 96-well polystyrene plates (TrueLine).

Bacterial growth was monitored by measuring the optical density at 600 nm of the culture at 2 h intervals using a plate reader (Infinite M200 PRO, TECAN). The cultures were grown in quadruplicate.

### Phages replication rate

Performance curves were used to quantify phage replication under different environmental conditions over a short incubation period (4 h). Replication of lytic phages CHOED and PvVp2 was performed according to the protocol described by Padfield et al. (2020), with some modifications. Briefly, the bacteria *V. anguillarum* and *V. parahaemolyticus* were grown overnight at 25 °C with shaking to achieve a cell density of 10^9^ cells. We diluted the bacterial culture to reach a concentration of 10^7^ UFC/mL, then we added 20 μL of phage (10^6^; MOI 0.02, N_0_) to each replicate. Microcentrifuge tubes were left static for 4 h at each condition (temperature, salinity, and pH), after which the phage was extracted using chloroform extraction. 100 μL of chloroform was added to 900 μL of culture, then the mixture was vortexed and centrifuged at 10,000 × *g* for 5 min. The supernatant was removed and placed in fresh Microcentrifuge tubes. Final phage titers (N4) were measured by plaque assay against the ancestral bacteria at 25 °C. Phage replication rate, r, was then calculated as *r* = ln(N4/N0)/4. These experiments were carried out in quadruplicate.

This assay has previously been used to evaluate the fitness or replication performance of phages under various environmental conditions (Knies et al., 2006, 2009). In this case, the low multiplicity of infection (MOI) used (0.02) minimizes the probability that a bacterial cell is initially infected by more than one phage, thereby reducing potential competition among phages and allowing replication performance to be evaluated under conditions with a low likelihood of multiple infections. Additionally, it has been documented that phages can exhibit longer latency periods when replicating under non-ideal conditions (Gnezda-Meijer et al., 2006). Therefore, using an incubation time of 4 h allows us to assess phage replication over extended replication periods while ensuring that phages replicating under optimal conditions can complete several cycles, maximizing the differences observed between ideal and non-ideal conditions. Moreover, under certain environmental conditions, some phages may become inactivated or adsorbed onto the host cell without completing their replication cycle. In these instances, our analysis may yield negative values. Thus, this assay was designed to evaluate phage performance under different environmental conditions and to contrast these results with the environmental conditions that allow the host bacterial replication, as determined by their specific growth constant.

The Temperatures used in this assay were 4 °C, 10 °C, 14 °C, 22 °C, 25 °C, 30 °C, 37 °C. For phage PvVp2, the experiments were also conducted at 40 °C and 45 °C. Salinities used were 0%, 0.85%, 2%, 3.5%, 5%, 7%, and 10% p/v. and the pH values used were 3, 4, 5, 6, 7, 7.7, 8, and 9. To evaluate the individual effect of each environmental factor, only one variable was modified in each experiment, while all other culture conditions were kept constant. Temperature assays were conducted at pH 7 and 3.5% (w/v) salinity; pH assays at 25 °C and 3.5% (w/v) salinity; and salinity assays at 25 °C and pH 7.

### One step

The one-step growth assay was performed following the protocol described by Hyman and Abedon (2009), under three different conditions of temperature, pH, and salinity. Based on the performance curve experiments, one-step growth assays were performed under selected temperature, pH and salinity conditions representing optimal and suboptimal phage replication parameters. These conditions were chosen to determine whether the reduced phage performance observed under non-optimal environmental conditions was associated with changes in the replication parameters. For *V. anguillarum*, the temperatures tested were 14°C, 25°C, and 30°C, while for *V. parahaemolyticus*, the temperatures were 14°C, 25°C, and 37°C. The pH conditions evaluated were 5, 7, and 9, and salinity conditions were 0.85%, 3.5%, and 7% w/v. For phage CHOED, samples for viral titration were collected every 5 minutes over 100 minutes to determine burst size and latent period. For phage PvVp2, samples were collected every 5 min for 60 min. Additionally, to achieve higher resolution during the lysis phase, samples were taken every 2 min for 20 min at 37 °C for *V. parahaemolyticus.*

In summary, 1 mL of a bacterial culture in exponential phase (10^7^ CFU/mL) was infected with 100 μL of the phage at an approximate multiplicity of infection (MOI) of 0.01. After 10 min of incubation, the mixture was centrifuged, re-suspended and diluted 25-fold in fresh culture medium. From that point on, a sample was removed every 5 min, and serial decimal dilutions were spotted on a previously prepared lawn of the host on agar plates. The plaques formed after overnight incubation were counted and plotted versus time to visualize the growth curve. These assays were performed in triplicate.

### Infection curves

Infection curves were used to evaluate the cumulative effect of phage infection on bacterial growth over 24 h under different temperatures. These assays were performed in 96-well plates to evaluate the infectivity of phage PvVp2 against *V. parahaemolyticus* and phage CHOED against *V. anguillarum*. Bacterial cultures were diluted in 1 mL of the LB broth (10^7^ cfu/mL) and inoculated with 100 μL of the phage stocks (10^8^ pfu/mL) and (10^9^ pfu/mL) to achieve two distinct multiplicities of infection (MOI) of 1 and 10, respectively. Wells were prepared in quadruplicate containing either the phage-host mixture, a positive bacterial growth control (without phage), or an LB broth negative control, to a final volume of 200 μL per well. To evaluate temperature impacts, plates were incubated in parallel at three controlled temperatures (14 °C, 25 °C, and 37 °C) for 24 h. Bacterial density was monitored by measuring optical density at 600 nm using an Infinite RM200 NanoQuant plate reader (TECAN). The area under the curve (AUC) for each condition was calculated according Xie et al. (2018), setting *Y* = 0 and with the following formula AUC = (AUC bacteria – AUC bacteria infected with phage)/AUC bacteria.

### Statistical analysis

Environmental tolerance and replication efficiency curves were analyzed using multiple independent *t*-tests per row (individual environmental levels). To formally account for heteroscedasticity across physiological extremes, unequal variances were corrected using Welch’s correction for each comparison. The inflation of Type I errors due to multiple testing was controlled via the False Discovery Rate (FDR) approach using the two-stage linear step-up procedure of Benjamini et al. (2006), setting a desired discovery threshold of *Q* = 5%. All statistical analyses were conducted using GraphPad Prism software (version 11.02).

Phage replication kinetics from one-step growth curves were modeled by non-linear regression using GraphPad Prism software (v11.0.2). Replicated data sets were fitted to a four-parameter sigmoidal model. To address experimental noise at early time points and achieve biological realism in latent period estimates, the baseline parameter (Bottom) was constrained by the initial viral titer of each assay. Curve comparison and statistical differences between environmental treatments were evaluated using a global fitting approach and assessed using an *F*-test (α = 0.05). Non-convergent datasets in which active viral replication was not observed, with R^2^ < 0.10, were excluded from mathematical modeling. This mathematical approach to resolving parameters from one-step growth curves follows established non-linear regression protocols for bacteriophage kinetic characterization (Mudgal et al., 2006; Litt and Jaroni, 2017).

Finally, to evaluate differences in bacterial growth kinetics, the Area Under the Curve (AUC) was calculated independently for each individual replicate and subsequently analyzed using an ordinary two-way ANOVA, with temperature and MOI as independent factors. Variance homogeneity was verified using Spearman’s test for heteroscedasticity. Post-hoc multiple comparisons were performed using Tukey’s test to determine specific pairwise differences. All statistical analyses and AUC calculations were conducted using GraphPad Prism software (version 11.0.2), with statistical significance set at α = 0.05.

## Results

### Isolation and characterization of phage PvVp2

The phage infecting *V. parahaemolyticus* CIBGEN003 was isolated from the hepatopancreas of Pacific white shrimp (*Penaeus vannamei*) with signs of AHPND (Ramírez-Sánchez et al., 2026), which was designated Vibriophage vB_Vp_PvVp2 according to Kropinski et al. (2009). After 48 h of incubation, phage vB_Vp_PvVp2 formed relatively small plaques (<2 mm) on *V. parahaemolyticus* at 30 °C. The titer of phage vB_Vp_PvVp2 was determined using log dilutions of the purified phage lysate, with a value of 2.3 × 10^9^ PFU per mL.

The PvVp2 genome was assembled into a single contig of 43,158 bp with an average sequencing coverage of 156×. Analysis using PhageTerm suggested the presence of terminal redundancy and circular permutation (Supplementary Material 2), a packaging strategy commonly observed in circularly permuted bacteriophages. Genome annotation identified 49 predicted open reading frames (ORFs), of which 26 (53.1%) encoded hypothetical proteins with no assigned function. Among the functionally annotated genes, a phage-encoded RNA polymerase located upstream of the structural gene cluster was identified. In addition, a putative auxiliary metabolic gene encoding a MazG-like pyrophosphatase was detected (Figure 1). Phylogenetic analysis revealed that PvVp2 clusters with other *Vibrio* infecting phages and shares nearly 100% nucleotide identity with phages vB_Vc_SrVc2 and vB_Vc_SrVc9. Based on genome-wide sequence similarity and phylogenetic placement, PvVp2 was classified within the class Caudoviricetes, order Autographivirales, family Autoscriptoviridae, and genus *Maculvirus* (Supplementary Figure 1).

**FIGURE 1:**
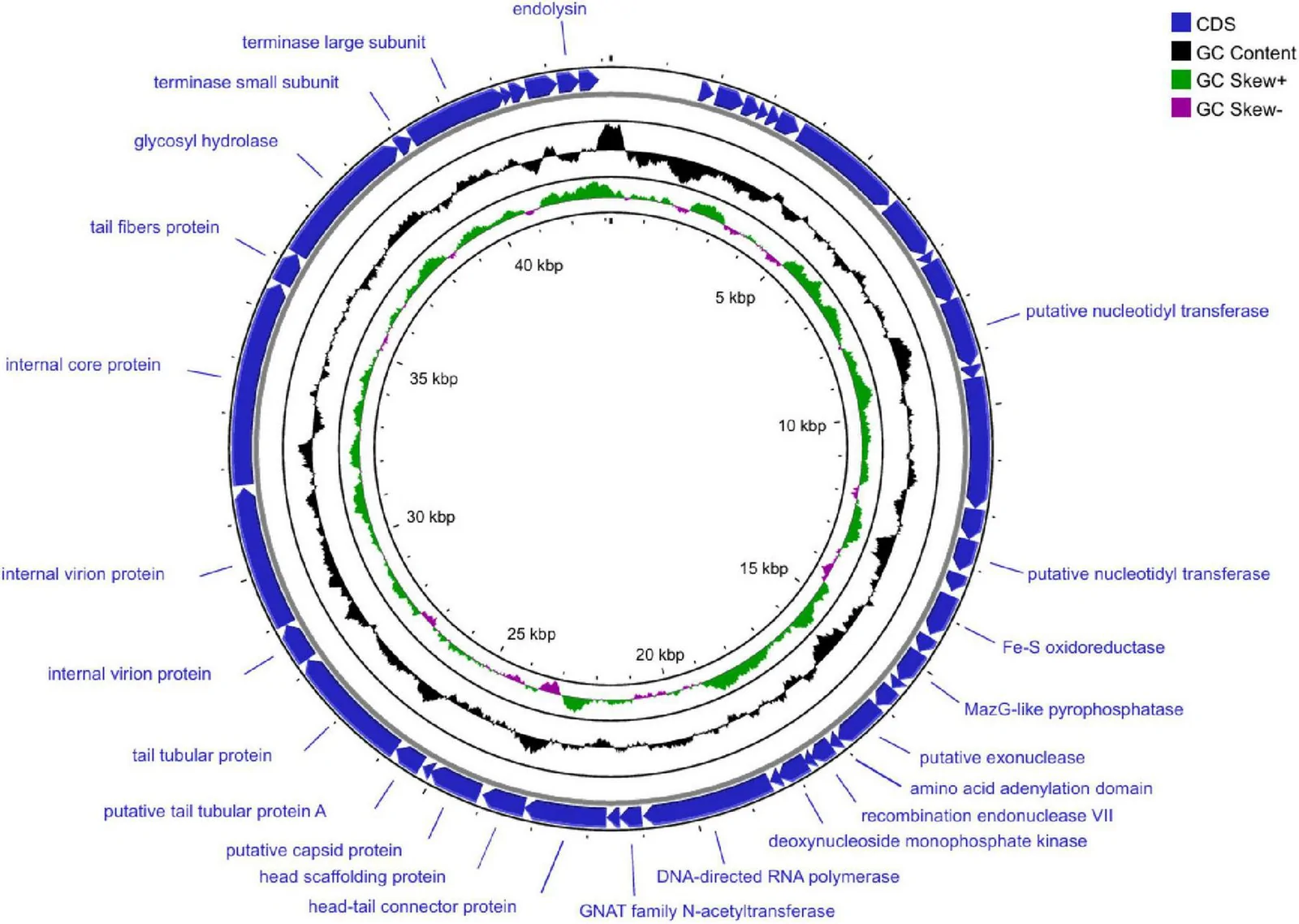
Genome of vibriophage PvVp2. The figure shows the different CDS with the GC content. Potential genes with predicted function are labeled. Hypothetical protein genes are not labeled.

### Performance curves of *Vibrio parahaemolyticus* and *V. anguillarum* under different environmental conditions

To understand how environmental conditions can affect the interaction between phages and vibrios, a first step is to examine how these conditions affect *Vibrio* growth. To evaluate this, we used phage PvVp2, which infects the pandemic strain of *V. parahaemolyticus* KX, and phage CHOED, which is specific to *V. anguillarum*. In this way, we also assessed the behavior of two different phages infecting bacteria adapted to distinct environmental conditions.

When evaluating the effect of temperature on the growth of these bacteria, it was observed that *V. parahaemolyticus* can grow at temperatures ranging from 15 °C to 45°C, with its highest specific growth rate at 37°C (Figure 2A). On the other hand, at most temperatures tested, *V. anguillarum* exhibited a lower specific growth rate than *V. parahaemolyticus* but was able to grow between 14°C and 40°C, with an optimal growth temperature of 25°C (Figure 2A). These results show that *V. anguillarum* is adapted to grow at lower temperatures than *V. parahaemolyticus*. In terms of maximum growth, *V. parahaemolyticus* achieves this at 30 °C and 37 °C, while at temperatures below 22°C, the culture does not even reach the stationary phase. *V. anguillarum*, on the other hand, reaches maximum growth at 25 °C, and at temperatures above 30°C or below 22°C, the observed growth is minimal (Supplementary Figure 2). These results show a similar pattern to that observed in the specific growth rates of these bacteria under the same conditions.

**FIGURE 2:**
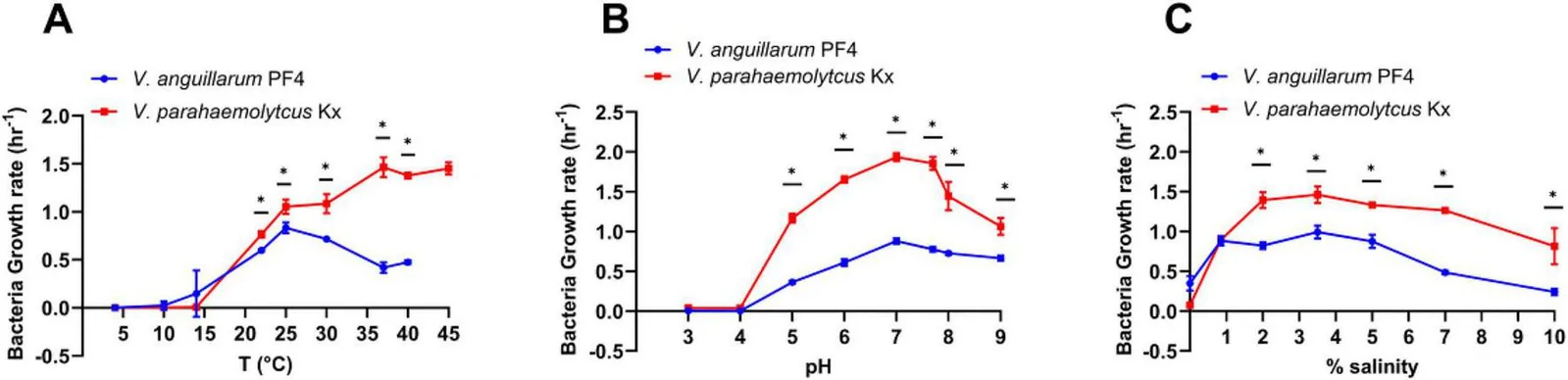
Bacterial growth rates of *V. parahaemolyticus* KX and *V. anguillarum* PF4 at different temperatures **(A)**, pH values **(B)**, and salinities **(C)**. Bacterial growth rates were calculated as the slope of ln(OD_600_) during the exponential growth phase. Bacteria were cultured in parallel in 96-well polystyrene plates, and OD_(600nm)_ was measured at 2-h intervals. Assays were performed in quadruplicate, and error bars represent standard deviations. Statistical comparisons were performed using multiple independent *t*-tests with Welch’s correction for unequal variances. Multiple comparisons were controlled using the two-stage linear step-up procedure, with a false discovery rate (FDR) threshold of *Q* = 5%. Significant differences are indicated by asterisks (*Q* < 0.05).

When evaluating the growth of these bacteria at different pH levels, it was observed that both exhibited similar behavior, growing between pH 5 and 9 (Figure 2B). As expected, both bacteria showed optimal growth at pH 7; throughout the entire range tested, *V. parahaemolyticus* exhibited a higher specific growth rate than *V. anguillarum*.

Using different salinity concentrations, a similar result was observed. Both bacteria were able to grow in salinity conditions ranging from 0% to 10% (Figure 2C). Vibrios are known to require salts for growth, so the low growth observed at 0% salinity is likely due to residual salts in the culture medium. Across all salinities tested, except for 0.85%, *V. parahaemolyticus* exhibited a higher specific growth rate than *V. anguillarum* (Figure 2C). As expected, for *V. anguillarum*, maximum growth was observed at pH 7, followed by pH 6 and pH 8, while for *V. parahaemolyticus*, no obvious differences were observed between pH 6 and 8. In terms of salinity, both bacteria showed maximum growth at 2% and 3.5% (Supplementary Figure 2). Taken together, these results show that *V. parahaemolyticus* is adapted to warmer temperatures than *V. anguillarum*, but in terms of pH and salinity, both species show similar tolerance patterns.

### Performance curves of phages PvVp2 and CHOED under different environmental conditions

One way to evaluate how environmental conditions affect the interaction between a phage and its host bacterium is to use performance curves, which show the ability of the phage to replicate in the bacterium under varying conditions. In this case, when assessing the replication capacity of phages PvVp2 and CHOED in their respective host bacteria at different temperatures, it was observed that phage PvVp2 replicated efficiently between 22°C and 40°C, decreasing at 45°C (Figure 3A). In contrast, phage CHOED replicated between 14°C and 30°C, with the latter as its optimal replication temperature. These results indicate that phage PvVp2 is adapted to replicate at higher temperatures than phage CHOED, whose activity decreased notably at 37°C. Conversely, phage PvVp2 lost activity at temperatures below 14°C (Figure 3A).

**FIGURE 3:**
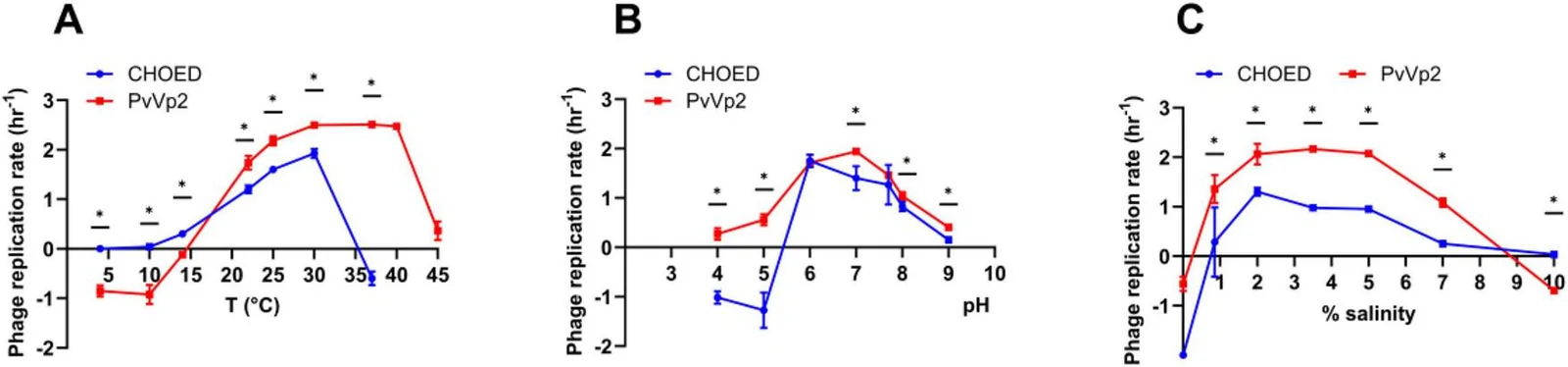
Phage replication performance of CHOED and PvVp2 at different temperatures **(A)**, pH values **(B)**, and salinities **(C)**. Phages were incubated with their respective host bacteria at an MOI of 0.02 for 4 h under different temperature, pH, and salinity conditions. Final phage concentrations were determined, and the replication rate was calculated as *r* = ln(N4/N0)/4. Assays were performed in quadruplicate, and error bars represent standard deviations. The detection limit was 100 PFU/mL. Statistical comparisons were performed using multiple independent *t*-tests with Welch’s correction for unequal variances. Multiple comparisons were controlled using the two-stage linear step-up procedure, with a false discovery rate (FDR) threshold of *Q* = 5%. Significant differences are indicated by asterisks (*Q* < 0.05).

When examined at different pH levels, phage PvVp2 exhibited a broader tolerance range, replicating between pH 4 and 9, with an optimum at pH 7 (Figure 3B). In contrast, phage CHOED reduced its activity at pH 4 and 5, reached its maximum replication at pH 6, and showed a steady decline up to pH 9.

The replication of the phages PvVp2 and CHOED was also affected by medium salinity. In this case, both phages replicated within a salinity range of 0.85%–7% (Figure 3C). Phage PvVp2 displayed optimal replication between 2% and 5% salinity, while phage CHOED’s replication was optimal at 2% and progressively declined at both lower and higher salinities (Figure 3C).

Taken together, these results demonstrate that the ability of a phage to replicate is influenced by environmental conditions. Phage PvVp2 is adapted to higher temperatures than phage CHOED and generally exhibits a broader tolerance range.

### Replication parameters of phages PvVp2 and CHOED under different environmental conditions

To understand the replication patterns observed in phages PvVp2 and CHOED under different environmental conditions, one-step infection curves were performed under varying conditions. This assay will allow us to determine the burst size and latency period of these phages under the different environmental conditions evaluated. The burst size of phage CHOED increased with temperature, reaching a maximum at 30 °C, which coincided with the highest observed replication rate (Figure 3A and Table 1). While no differences in burst size were observed between 14 °C and 25 °C, the latency period at 14 °C was twice that observed at 25 °C, which could explain the lower replication rate at the lower temperature (Table 1 and Supplementary Figure 3). For phage PvVp2, no latency period or burst size could be observed at 14 °C. However, at 25 °C, a 10 min latency period and a burst size of 8.7 PFU/cell were observed, indicating shorter replication periods than those of phage CHOED but with lower viral particle production. At 37 °C, it was difficult to determine the latency period of this phage when samples were collected every 5 min; the one-step assay showed an immediate and sustained increase in PFU/cell, suggesting an extremely short latency period. When performing the one-step growth curve with samples taken at shorter intervals, we observed that PFU/cell began to increase at 2 min, confirming that this phage has a very short latency period (Table 1 and Supplementary Figure 4).

**TABLE 1:** Replication parameters of phages PvVp2 and CHOED under different environmental conditions.

Phage	Parameter	Temperature				pH			% salinity		
		14 °C	25 °C	30 °C	37 °C	5	7	9	0.85%	3.5%	7%
CHOED	Burst size (PFU/cell)	33.4	33	44.8	ND	8.3	33	–	8.8*	33	–
	Latent period (min)	50	25	15	ND	45	25	–	45*	25	–
PvVp2	Burst size (PFU/cell)	–	8.7	ND	11.1	–	8.7	–	–	8.7	–
	Latent period (min)	–	10	ND	≤2	–	10	–	–	10	–

ND: Not determined. –: No detectable burst size or latent period.

*: Obtained from one-step modeled curve.

At pH 9, it was not possible to observe a latency period or burst size for either phage, consistent with a low replication rate for both phages under this condition (Figure 3). At pH 7, considered the control condition, the parameters correspond to those observed at 25 °C, the temperature control condition. At pH 5, only the latency period and burst size of phage CHOED were detectable. In this case, an extended latency period was observed compared to the control condition, along with a decrease in the number of phages released, indicating that although replication occurs, this pH condition negatively affects it (Table 1).

Finally, when performing one-step infection curves at different salinities, it was observed that the burst size and latency period of both phages could be determined only at 3.5% salinity, which corresponds to the standard condition under which the assays were performed (Table 1). These findings help explain the differences in replication levels observed for each phage under the environmental conditions tested. Variations in temperature, pH, and salinity not only influence bacterial growth but also affect phage replication dynamics, as reflected by changes in burst size and latent period. Consistent with the performance curves, phage PvVp2 exhibited higher replication parameters (higher burst size and shorter latency period) at elevated temperatures than phage CHOED. This pattern suggests that PvVp2 is better adapted to replicate under warmer conditions.

Statistical analysis of burst size from the different one-step experiments did not show statistical significance due to the high biological variability in this kind of experiment. Therefore, the phage replication kinetics from one-step growth curves were modeled using a four-parameter sigmoidal model fitted by non-linear regression. As expected, data from experiments at pH 9 or salinity 7% in which no phage replication was observed did not fit the model and were excluded (R^2^ < 0.10) (Supplementary Figure 5). Interestingly, the modeling enabled visualization of the one-step curve for phage CHOED at a salinity of 0.85%, yielding a burst size of 8.8 and a latency period of 45 min. The statistical analysis using a global curve comparison via the extra sum-of-squares *F*-test showed that the curves differed significantly (*P* < 0.0001), confirming that these environmental conditions affect the replication of this phage. For phage PvVp2, it was only possible to model the curve at 25 °C, pH 7, and 3.5% salinity, and the second curve at 37 °C, which was performed with shorter sampling times, suggesting an extremely short latency period (≤2 min).

### Infection of phages PvVp2 and CHOED under different temperatures

The differences in the performance curves of the PvVp2 and CHOED phages at various temperatures led us to evaluate infection curves at 14 °C, 25 °C, and 37 °C for both phages and their respective hosts. This assay will allow us to evaluate the ability of phages to lyse the bacterial culture under the tested environmental conditions. Phage infectivity was assessed using the area under the curve (AUC) method. Overall, the CHOED phage demonstrated greater infectivity against its host bacterium at both 14 °C and 25 °C, and this did not change significantly when the multiplicity of infection (MOI) was increased from 1 to 10 °C at 25 °C (Figure 4 and Supplementary Figure 5). However, at 37 °C, the CHOED phage failed to lyse the *V. anguillarum* culture efficiently at an MOI of 1, and lysis was observed only at an MOI of 10. This result contrasts with the performance curve, which indicated that this phage could not replicate at that temperature (Figures 3, 4, and Supplementary Figure 6). In contrast, the PvVp2 phage exhibited its highest lysis of the *V. parahaemolyticus* culture at 25 °C, with a reduction in lysis observed at both 14 °C and 37 °C (Figure 4 and Supplementary Figure 6). Unlike the CHOED phage at 37 °C, the PvVp2 phage showed no variation in its ability to lyse the *V. parahaemolyticus* culture when the MOI was adjusted from 1 to 10.

**FIGURE 4:**
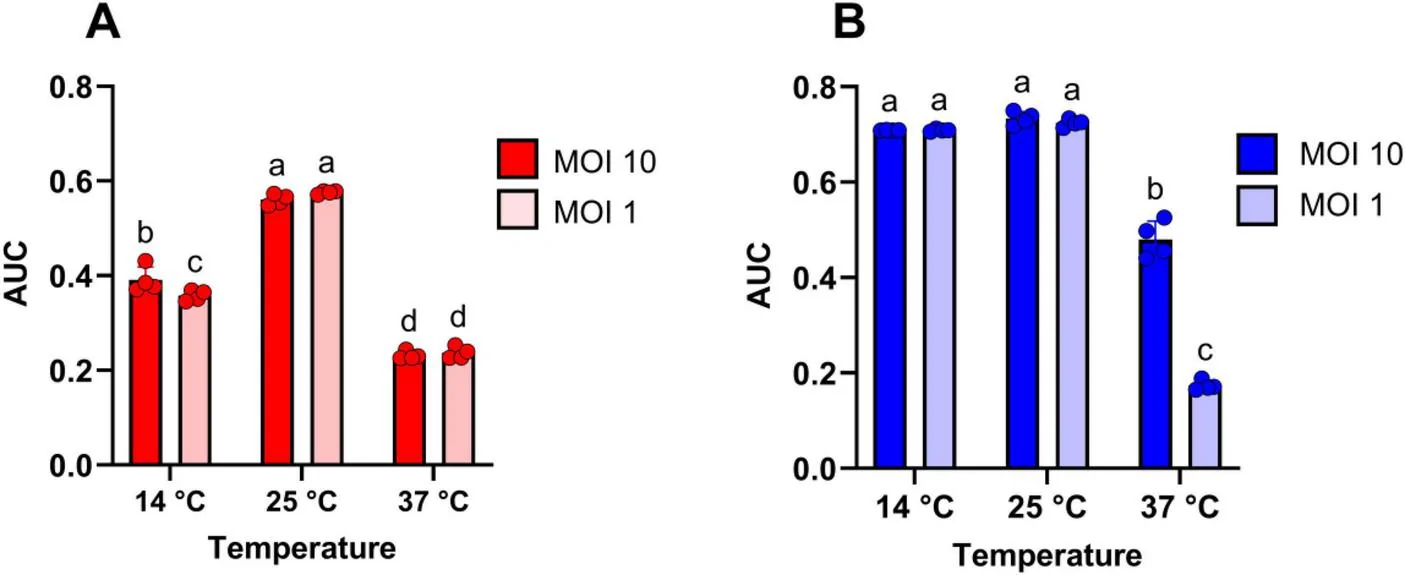
Infection curves at different temperatures. Area under the curve (AUC) values obtained from infection curves with phages **(A)** PvVp2 and **(B)** CHOED and their respective host bacteria, *V. parahaemolyticus* and *V. anguillarum*, under different temperatures and MOIs. Infection curves were performed in four replicates, and error bars represent standard deviations. Statistical differences in AUC values were assessed using a two-way ANOVA, with temperature and MOI as independent factors. Homogeneity of variance was assessed using Spearman’s test for heteroscedasticity, and Tukey’s test was used for post-hoc multiple comparisons. Statistical significance was set at α = 0.05.

Overall, these findings suggest that temperature significantly influences the replication characteristics of these phages, thereby affecting their ability to lyse their host bacterial cultures. Furthermore, bacteria seem to be better adapted to a broader range of environmental conditions than the bacteriophages that infect them. This study provides a basis for projecting how the interaction between phages and bacteria in the genus *Vibrio* could be altered under changing environmental conditions.

## Discussion

In this study, we report the isolation and characterization of phage PvVp2 and assess the effects of various environmental conditions on its replication and lysis on *Vibrio parahaemolyticus*, in comparison with phage CHOED and its host bacterium, *Vibrio anguillarum*. The results indicate that the range of environmental conditions that sustain phage replication is narrower than the range that allows bacterial growth. Furthermore, similar to its host bacterium, phage PvVp2 appears to be better adapted to replicate on its host under warmer environmental conditions than phage CHOED. However, these differences are not entirely translated to infection curves.

Phylogenetic analysis of phage PvVp2 indicated it belongs to the *Maculvirus* genus, and revealed a close relationship with other vibriophages, including phage VP93. This phage, specific to *V. parahaemolyticus*, is characterized by the presence of an RNA polymerase gene located at the end of the module associated with nucleic acid metabolism and was initially isolated from southern Chile (Bastías et al., 2010). Phage VP93 has been described as cosmopolitan, as highly similar phages have also been isolated from the coasts of Mexico. Therefore, the identification of additional, closely related phages from these regions is not unexpected. In this context, it would be of particular interest to evaluate in the future the performance of two phylogenetically related phages isolated from warm and cold environments under different temperature conditions.

Genomic analysis of phage PvVp2 revealed the presence of a gene encoding a MazG-like pyrophosphatase. This gene has been identified as an auxiliary metabolic gene (AMG) in phages, where it may function as a pyrophosphohydrolase involved in the metabolism of dGTP and dCTP, thereby contributing to host genome degradation during infection (Rihtman et al., 2019). Alternatively, other authors have proposed that such genes may regulate second messenger levels, preventing programmed cell death in bacteria infected by the phage (Fan et al., 2022). Overall, the presence of this gene likely contributes to the successful progression of phage PvVp2 infection.

A notable aspect of this work is that it evaluates the impact of environmental conditions on phage replication using performance curves, their associated replication parameters and infection curves. Typically, studies characterizing new phages focus on assessing phage stability and viability under different environmental conditions (particularly temperature and pH) (Flores et al., 2020; Yang et al., 2023). However, such studies do not necessarily capture how these conditions influence the phage’s ability to replicate and interact with its host bacteria. In this context, the use of performance curves provides a more accurate assessment of the effects of environmental conditions on phage biology. In this context, previous studies have demonstrated that phages can remain active across a broader temperature range than their host bacteria (Mojica and Brussaard, 2014). This property is likely attributable to their protein-based structure, which tends to be more thermally stable than cellular membranes. In contrast, the temperature range that supports phage replication is generally narrower than that which allows continued bacterial growth (Mojica and Brussaard, 2014). The present study corroborates this observation, showing that the environmental range supporting phage replication was narrower than the range supporting bacterial growth. Phage CHOED replicated up to 30 °C, whereas its host, *V. anguillarum*, continued to grow, although at a reduced rate, at 37 °C. A similar pattern was observed for phage PvVp2, which retained replicative capacity up to 45 °C, albeit at lower levels than at 37 °C or 40 °C, while its host, *V. parahaemolyticus*, continued to grow efficiently at 45 °C.

Both bacteria grew at pH 5 or higher, whereas phage replication occurred over a similar pH range, except for phage CHOED, which failed to replicate at pH 5. This inability to replicate under acidic conditions is likely due to viral particle inactivation, as reported in several studies (Ren et al., 2022; Cai et al., 2023). Regarding salinity, previous studies have shown that although phage particles can remain stable over a broader range of salinities than their hosts, phage replication and, particularly, adsorption to host cells may still be affected (Kukkaro and Bamford, 2009; Mojica and Brussaard, 2014). Consistent with this, the salinity range supporting bacterial growth was slightly broader than the range that allows phage replication, with CHOED being the phage most affected by salinity variations. The broad environmental tolerance observed in both bacteria and phages likely reflects their adaptation to marine environments.

Regarding the effect of environmental conditions on replication kinetic parameters, deviations from optimal conditions negatively affect phage replication, resulting in longer latent periods and lower burst sizes (Zaburlin et al., 2017; Briggiler Marcó et al., 2019). Similar results were observed in this study, where at 14 °C the latent period of phage CHOED extended to approximately 50 min, whereas for phage PvVp2 replication parameters could not be determined. Among the three environmental variables evaluated, salinity appears to have the greatest impact on phage replication, as replication kinetic parameters could be determined at 3.5% salinity and 0.85% for phage CHOED, where replication parameters were severely affected. These results highlight the critical role of salinity in the infectivity of these phages (Kukkaro and Bamford, 2009).

An interesting point is that the impact of temperature on performance curves does not always align with that observed in infection curves. Although both approaches evaluate the phage–host interaction, they provide complementary information. Performance curves evaluate the ability of the phage to replicate under specific environmental conditions during a short incubation period, whereas infection curves evaluate the effect of phage infection on bacterial growth over a longer period (24 h). Therefore, the results obtained from these assays may not necessarily show the same pattern, as they reflect different aspects and time scales of the phage–host interaction. To address this complexity, numerous mathematical models have been developed to describe interactions between bacterial and phage populations, each focusing on different parameters, such as specific bacterial growth rate, burst size, or latency periods (Cairns et al., 2009; García et al., 2019; Botelho et al., 2021). Additionally, software has been created to model and visualize these phenomena (Nilsson, 2022). In this study, at 14 °C the CHOED phage shows a significant reduction in replication compared with 25 °C. However, its ability to lyse bacteria does not vary significantly between these temperatures. The analysis of the replication parameters of this phage at the two temperatures reveals that the latency period doubles at 14 °C compared with 25 °C. This increase in latency affects the thermal performance assay, which is conducted over 4 h, while its impact on a 24 h infection curve is likely less pronounced. The high lytic capacity of CHOED at 14 °C may also be influenced by the low growth of *V. anguillarum* at that temperature, which reduces the ability of this bacterium to recover from phage predation. At 37 °C, CHOED shows reduced lysis on the host bacterium, but only with an MOI of 10. In this case, the high concentration of phages could lead to bacterial lysis, even with low phage replication efficiency, a possibility that could be further enhanced by phage adaptation after several replication cycles (Kering et al., 2020; Greenrod et al., 2026). On the other hand, the phage PvVp2 maintains lytic activity across different temperatures; however, this capacity decreases by approximately half at 14 °C and 37 °C, irrespective of the multiplicity of infection (MOI) used. It is important to note that lysis of *V. parahaemolyticus* still occurs at 14 °C, although the phage’s ability to replicate decreases significantly compared to 25 °C, and the replication parameters at that temperature remain undetermined. The discrepancy between these results may be attributed to a potential adaptation of the phage to the given temperature during the infection assay, or to limitations in detecting differences in the one-step and performance curve assays. Regardless, the findings indicate that infection curves reflect a complex interplay between phages and their host bacteria. As with other cases, this interaction is also influenced by environmental factors, such as temperature. Gaining insight into how these environmental variables affect specific aspects of phage and bacterial replication can deepen our understanding of this intricate phenomenon.

Considering the findings of this study alongside those reported by other researchers, it becomes evident that we must acknowledge the potential influence of environmental variables on the efficacy of phage therapy, particularly in aquaculture targeting pathogenic vibrios. In this context, factors such as salinity and pH have been shown to significantly impact the success of phage treatments (Silva et al., 2014; Choudhury et al., 2019). Given the natural variability in marine and aquaculture production conditions (Kalatzis et al., 2018), it is crucial to investigate how environmental factors influence phage replication and infectivity.

Differences in growth and replication capacity likely reflect the environmental conditions from which these strains were isolated. *V. parahaemolyticus* and phage PvVp2 appear better adapted to warmer environments, whereas *V. anguillarum* and phage CHOED are suited to colder waters, such as those of southern Chile. Similar patterns have been observed in phages from diverse environments (Yu et al., 2006; Luhtanen et al., 2018; Gil et al., 2021; Demina et al., 2022), raising the question of how climate-driven changes could alter bacteriophage–host interactions (Danovaro et al., 2011). Although this study did not directly evaluate the effects of climate change, it provides a baseline for understanding how environmental conditions may influence interactions between vibrios and their phages.

## Conclusion

These results show that the phage PvVp2 is adapted to warmer environments, exhibiting a broader temperature tolerance than phage CHOED. Despite this adaptability, the range of environmental conditions supporting the replication of both phages is narrower than that of their host bacteria. Interestingly, the ability of these phages to partially lyse their bacterial hosts appears to occur over a wider temperature range than their replication does, highlighting the complexity of this phenomenon. These findings underscore the significant impact of temperature and salinity on phage replication, offering insight into how environmental alterations may influence interactions between vibrios and their phages.

## Data Availability

All data generated in this study are available at figshare.com with the following DOI: https://doi.org/10.6084/m9.figshare.31852531.

## Footnotes

1
https://phage.usegalaxy.eu/
