# Supplementary material for: Effect of environmental conditions on two vibriophages and their host
Source: Front Microbiol. 2026 Oct 1;17:1914226. doi: 10.3389/fmicb.2026.1914226 (PMC13671069; doi:10.3389/fmicb.2026.1914226)
Supplement: Supplementary file 1 [file Data_Sheet_1.pdf]

Running head: Vibriophage environmental performance

Impact of environmental conditions on the performance of vibriophages.

Marcela León[1‡], Neri Vargas[1‡], Kívely Lozano[1], Santiago Gacitúa[1], Carlos Omar Lomelí-Ortega[2], Irais Ramírez-Sánchez[2], Eduardo Quiroz[2] and Roberto Bastías[1*]

[1] Instituto de Biología, Pontificia Universidad Católica de Valparaíso, Valparaíso, Chile.

[2] Centro de Investigaciones Biológicas del Noroeste S.C. Av., Instituto Politécnico Nacional 195, Col. Playa Palo de Santa Rita Sur, La Paz 23096, Baja California Sur, México

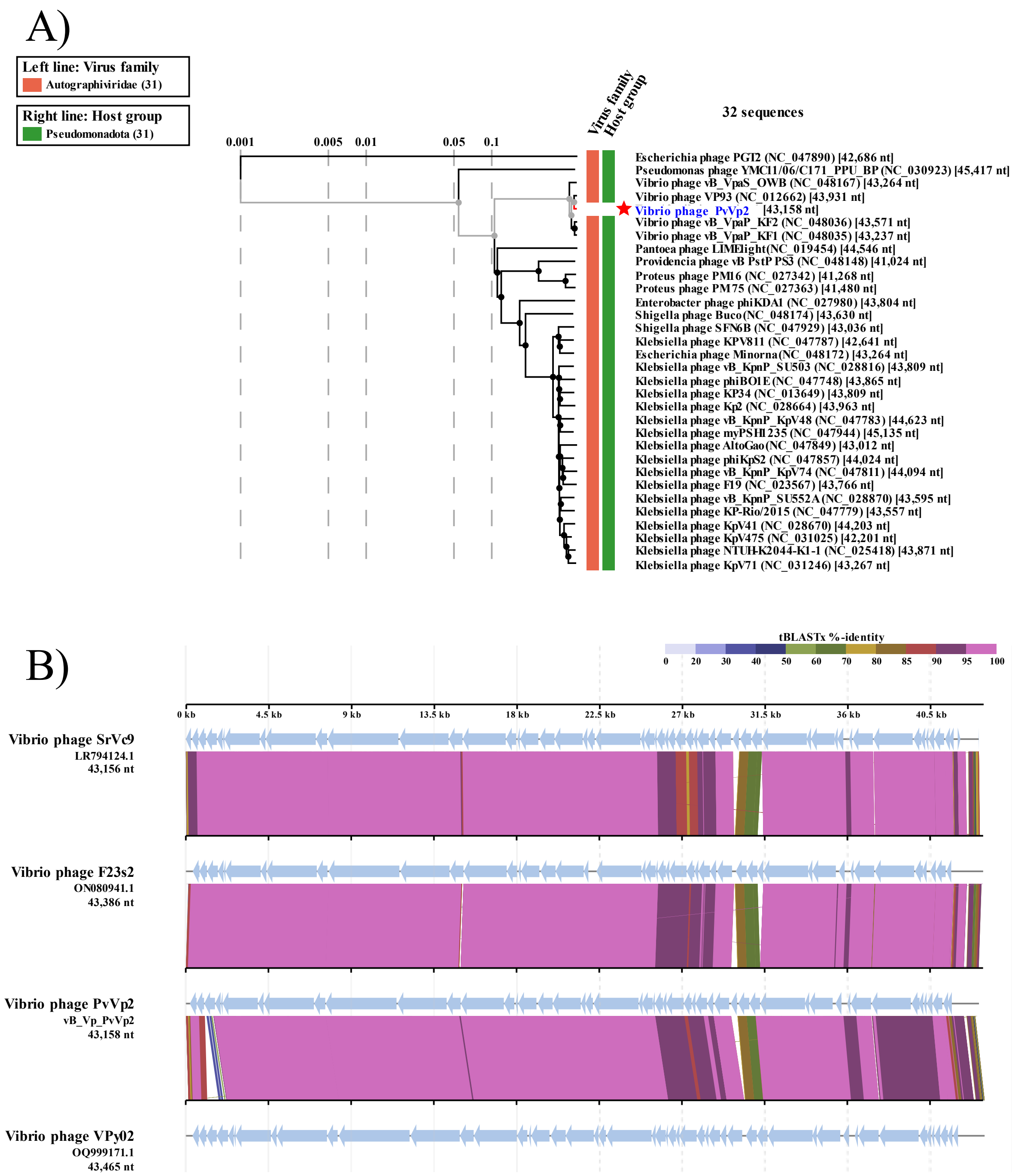

Supplementary Figure 1. Phylogenetic and Genomic Analysis of Phage PvVp2. (A) Viral proteomic tree showing the taxonomic classification of phage PvVp2 among other known bacteriophages, highlighting its clustering with other Vibrio phages. (B) Whole-genome alignment between phage

PvVp2 and its most closely related reference phages, showing high nucleotide identity and conserved gene synteny

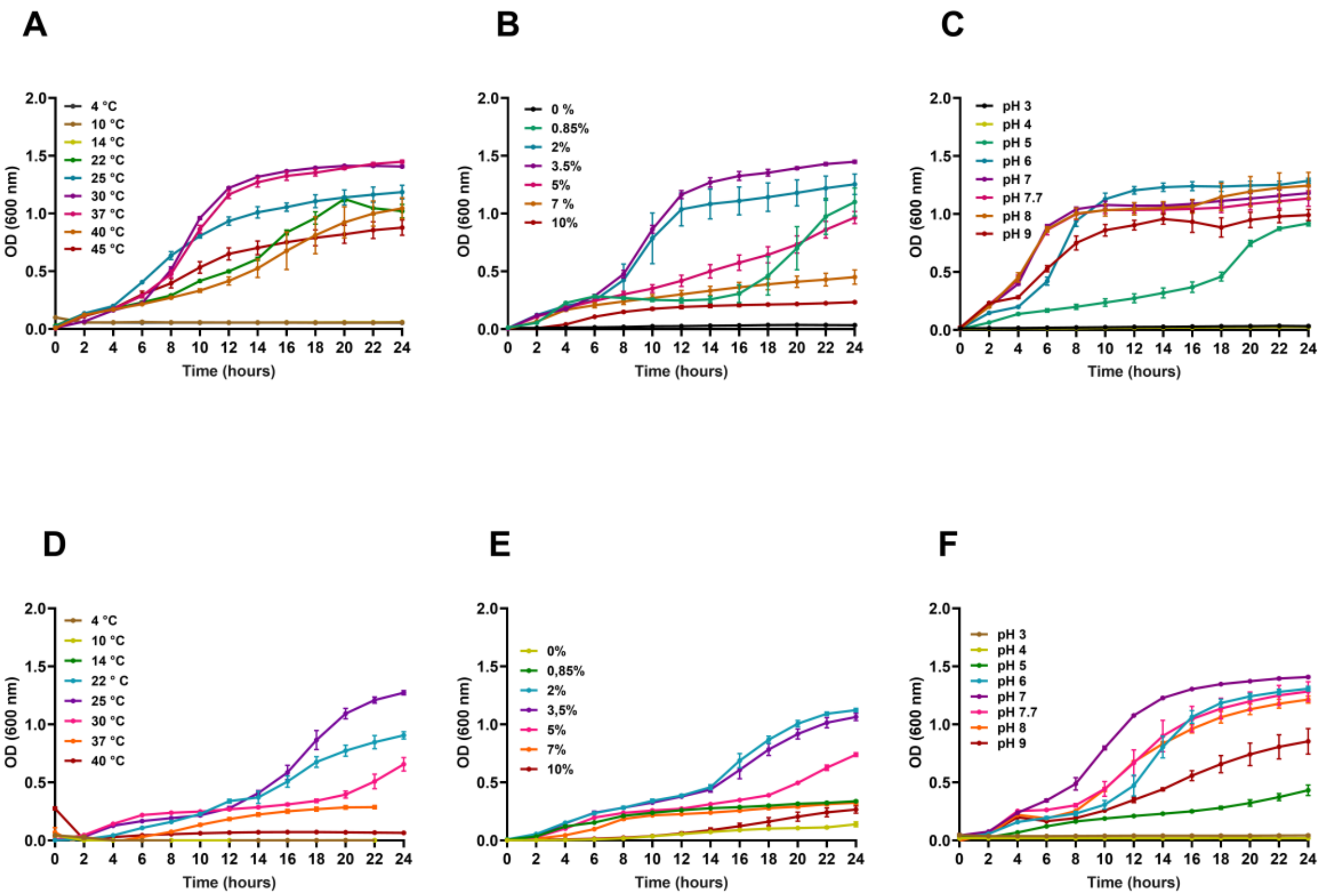

Supplementary Figure 2. Growth curves of *Vibrio parahaemolyticus* Kx and *Vibrio anguillarum* PF4 under varying environmental conditions. Total growth (OD 600 nm) measured over 24 hours for *V. parahaemolyticus* (A-C) and *V. anguillarum* (D-F) across a range of temperatures (A, D), salinity percentages (B, E), and pH levels (C, F). Error bars represent standard deviation from quadruplicate assays.

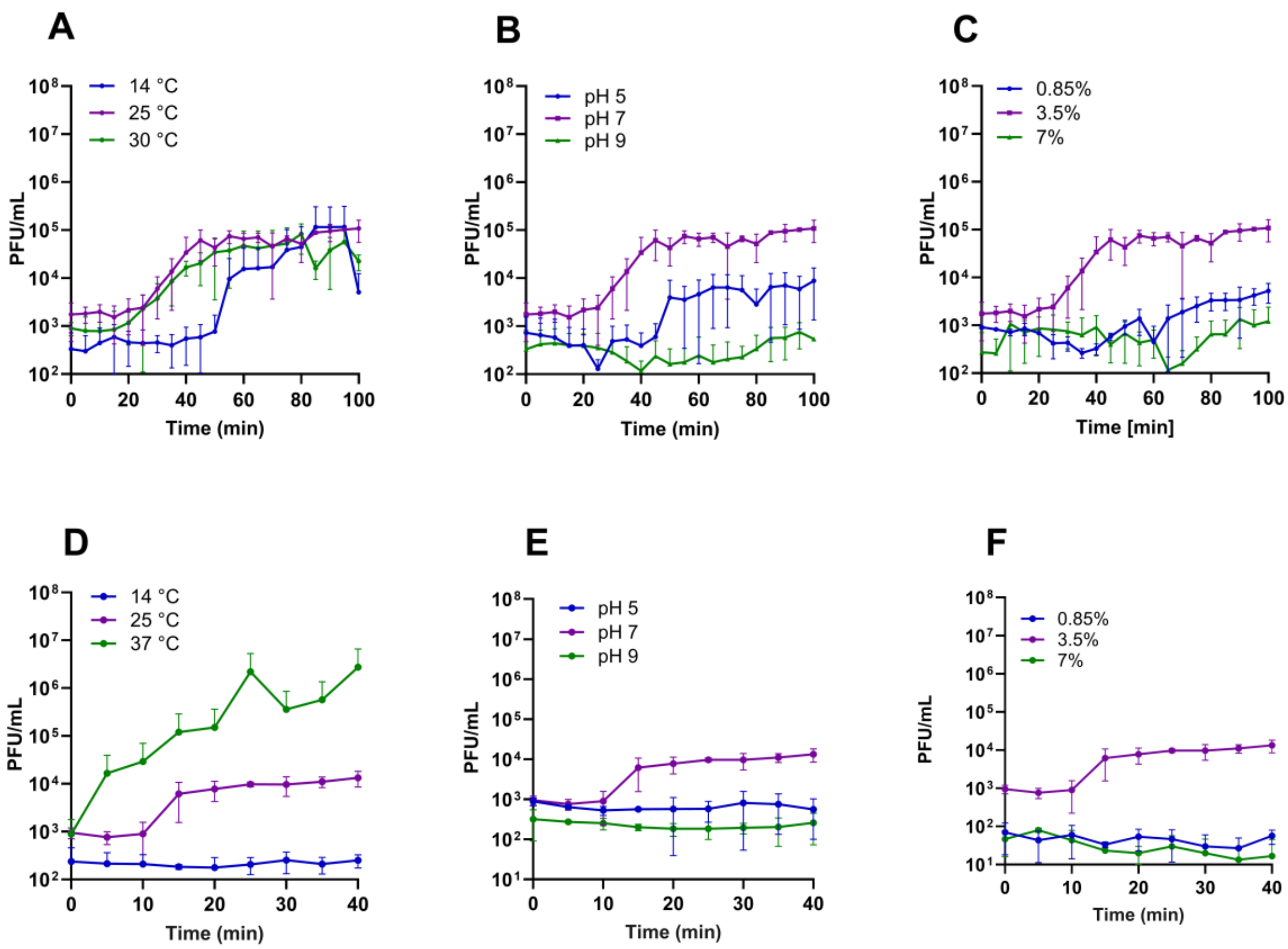

Supplementary Figure 3. One-step growth curves of phages CHOED and PvVp2 under different environmental stressors. The figure illustrates the replication kinetics (PFU/mL over time) for the *V. anguillarum* phage CHOED (A-C) and the *V. parahaemolyticus* specific phage PvVp2 (D-F) at various temperatures (A, D), pH levels (B, E), and salinities (C, F). These curves were used to calculate the burst size and latent periods reported in the study.

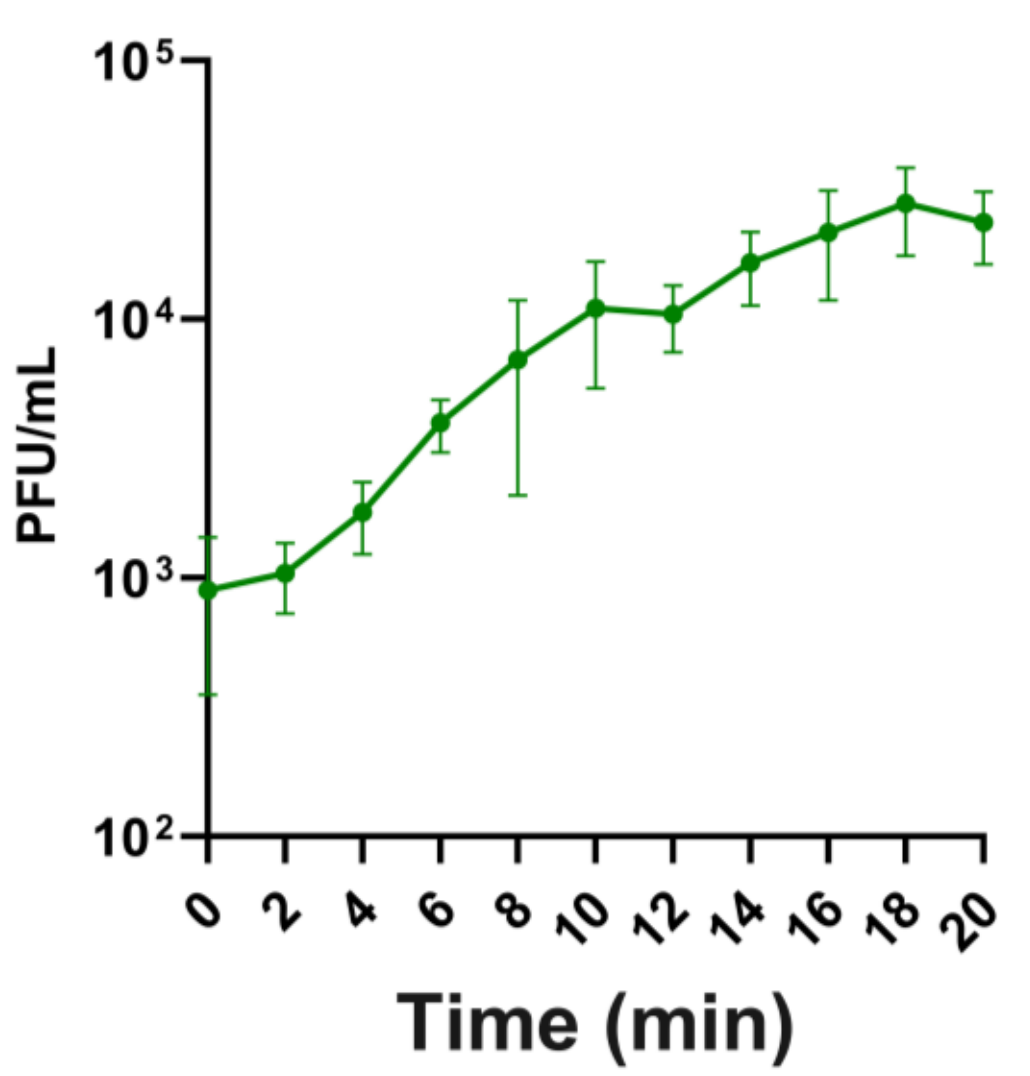

Supplementary Figure 4. One-step growth curves of phage PvVp2. Replication kinetics of phage PvVp2 infecting *V. parahaemolyticus*. Sampling at 2-minute intervals to resolve the extremely short latent period observed at 37°C.

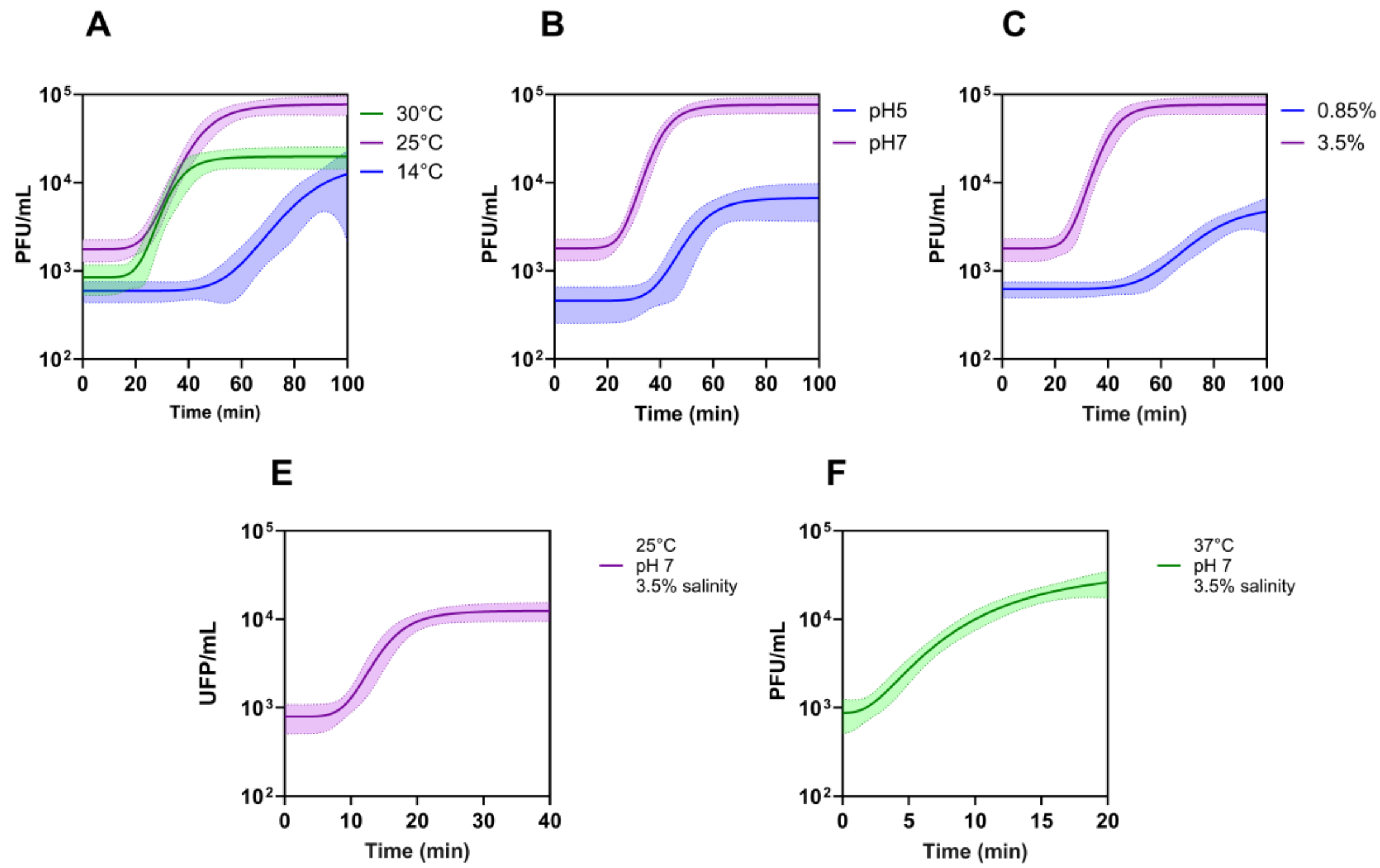

Supplementary Figure 5. **Modeled one-step growth curves**. Panels A, B, and C display the replication curves of phage CHOED infecting *Vibrio anguillarum* under varying conditions of temperature (A), pH (B), and salinity (C). Panels E and F show the replication kinetics of phage PvVp2 infecting *Vibrio parahaemolyticus* at pH7, 3.5% salinity and 25°C (E) and 37 °C (F). Lines represent the best-fit non-linear regression curves modeled via a four-parameter sigmoidal equation. Shaded areas around the curves represent the 95% asymmetrical confidence bands. Inhibitory environmental conditions resulting in flat replication profiles and non-convergent mathematical models ($R^2 < 0.10$) were excluded from graphic representation.

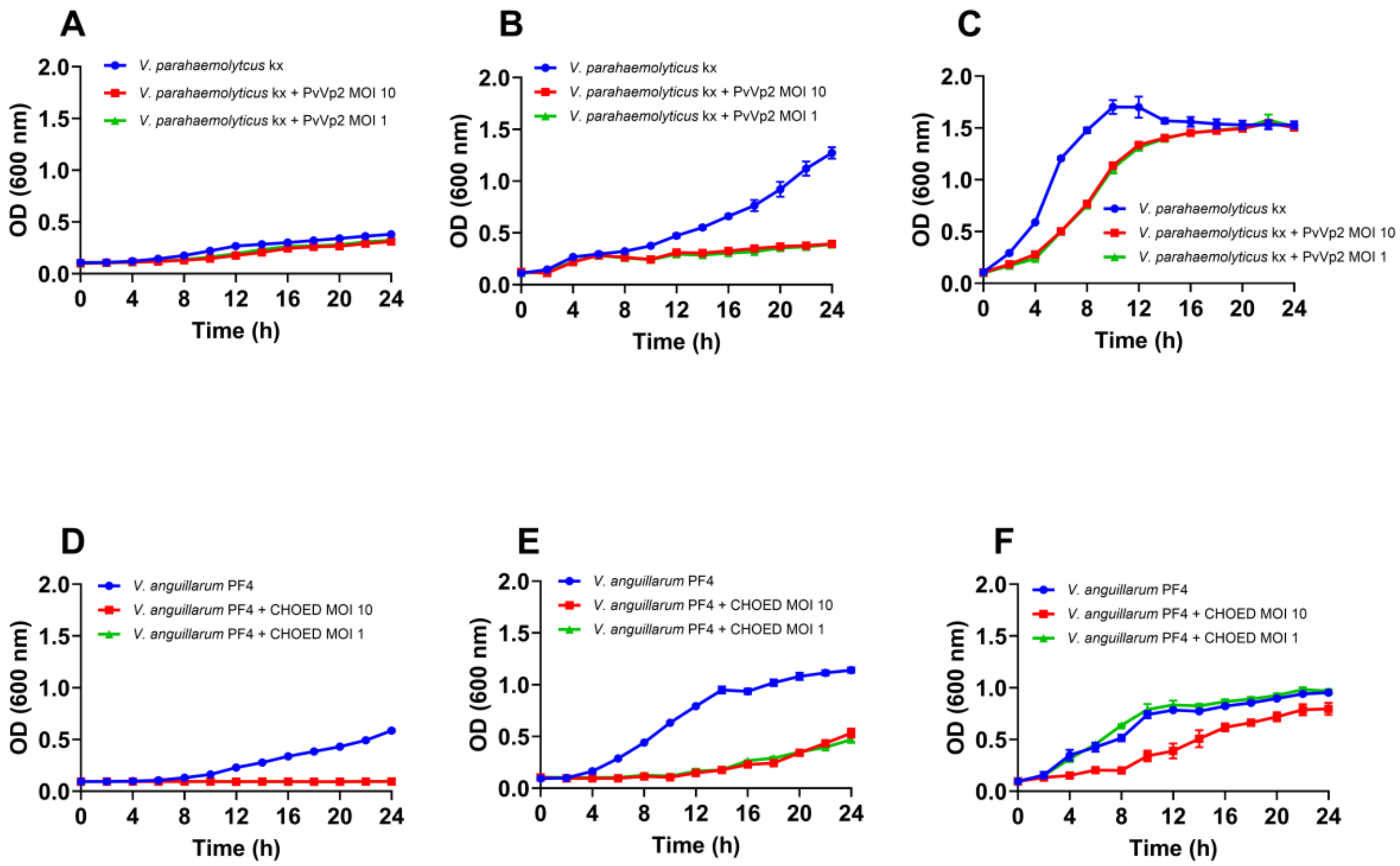

Supplementary Figure 6. Infection curves at different temperatures. The figure shows the infection of *V. parahemolyticus* with the phage PvVp2 (A-C) and *V. anguillarum* with the phage CHOED (D-E) at multiplicities of infection of 10 and 1**.** The infection curves were performed at 14 °C (A and D), 25 °C (B and E), and 37 °C (C and F). Bacterial growth was monitored by measuring the optical density at 600 nm. The experiment was performed in four replicates, and the error bars represent the standard deviation.
